# Case report: proximal tubule impairment following volatile anesthetic exposure

**DOI:** 10.14814/phy2.12560

**Published:** 2015-09-28

**Authors:** Evan C Ray, Khaled Abdel-Kader, Nicholas Bircher, Helbert Rondon-Berrios

**Affiliations:** 1 Renal-Electrolyte Division, University of Pittsburgh Medical CenterPittsburgh, Pennsylvania; 2 Division of Nephrology and Hypertension, Vanderbilt UniversityNashville, Tennessee; 3 Department of Anesthesiology, University of Pittsburgh Medical CenterPittsburgh, Pennsylvania

**Keywords:** Desflurane, glucosuria, glycosuria, inhalation anesthetic, isoflurane, kidney function, phosphaturia, proximal tubule, renal tubular acidosis, sevoflurane, type II RTA, volatile anesthetic

## Abstract

The safety of contemporary volatile anesthetic agents with respect to kidney function is well established, and growing evidence suggests that volatile anesthetics even protect against ischemic nephropathy. However, studies examining effects of volatile anesthetics on kidney function frequently demonstrate transient proteinuria and glycosuria following exposure to these agents, although the cause of these findings has not been thoroughly examined. We describe the case of a patient who underwent a neurosurgical procedure, then experienced glycosuria without hyperglycemia that resolved within days. Following a second neurosurgical procedure, the patient again developed glycosuria, now associated with ketonuria. Further examination demonstrated nonalbuminuric proteinuria in conjunction with urinary wasting of phosphate and potassium, indicative of proximal tubule impairment. We suggest that transient proximal tubule impairment may play a role in the proteinuria and glycosuria described following volatile anesthetic exposure and discuss the relationship between these observations and the ability of these agents to protect against ischemic nephropathy.

## Introduction

The number of major surgeries per year exceeds 230 million world-wide (Weiser et al. 2008). Many of these procedures require general anesthesia, typically including volatile anesthetics. Although use of historic volatile anesthetics was associated with nephrotoxicity (Mazze 2006), contemporary anesthetic agents, such as sevoflurane, isoflurane, and desflurane, are generally regarded as safe with respect to kidney function on the basis of numerous studies showing no effect on creatinine clearance (Eger et al. 1997a; Eger et al. 1997b; Kharasch et al. 1997; Higuchi et al. 1998; Nishiyama et al. 1998; Goldberg et al. 1999; Groudine et al. 1999; Ebert and Arain 2000; Obata et al. 2000; Higuchi et al. 2001; Kharasch et al. 2001; Conzen et al. 2002; Ebert et al. 1998). Additionally, a growing body of literature indicates that volatile anesthetics are protective against ischemic nephropathy (Cai et al. 2014; Fukazawa and Lee 2014).

The nephrology service at our hospital was consulted for unexplained glycosuria without elevated serum glucose in a patient who underwent a series of major neurosurgical procedures. Further evaluation of this patient revealed hypophosphatemia, urinary phosphate wasting, proteinuria without albuminuria, ketonuria, and inappropriately elevated urinary potassium loss, suggesting proximal tubule dysfunction. Lacking other explanations for these findings, we reviewed the literature for evidence that volatile anesthetics may affect proximal tubule dysfunction. Numerous studies confirm that contemporary volatile anesthetics have no adverse effects on glomerular filtration, however, glycosuria and proteinuria following volatile anesthetic exposure has been noted in the anesthesiology literature to be “extremely common” (Kharasch et al. 2001), as is elaboration of urinary biomarkers suggestive of transient proximal tubule impairment (reviewed below). After presenting the clinical course of our patient, we briefly review clinical data pertinent to the effects of volatile anesthetics on proximal tubule function and speculate about the apparent association between these agents and proximal tubule function.

## Case Report

**Figure 1:**
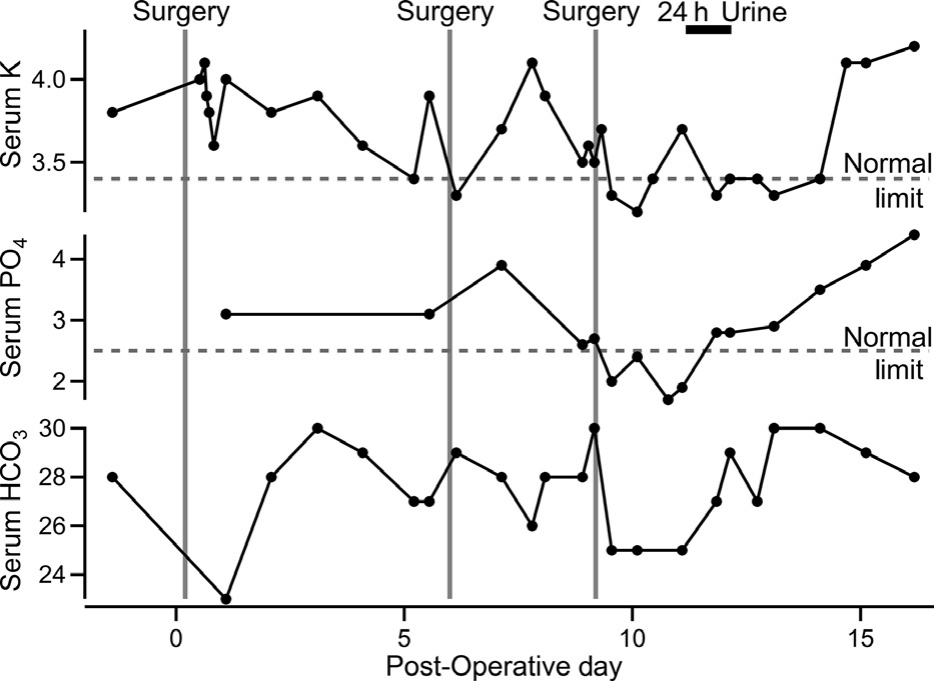
Serum potassium, phosphate, and bicarbonate relative to surgeries and timing of 24-h urine collection. Dashed lines represent lower limit of normal for each electrolyte at the treating hospital’s chemistry laboratory. Lower limit of normal for bicarbonate (21 mmol/L) is not shown. Vertical lines represent initiation of surgery on days zero, six, and nine. Units are as follows: potassium, mEq/L; phosphate, mg/dL; and bicarbonate, mmol/L.

A patient in the fourth decade of life with no other medical history and no known family history of kidney disease was admitted for resection of an olfactory tumor. After induction with midazolam, fentanyl, lidocaine, and propofol, maintenance of anesthesia consisted of infusions of remifentanyl and propofol, and desflurane with fresh gas (oxygen/air) flow rates of 2 L/min and concentrations of 5.0–7.3%. Rocuronium provided neuromuscular blockade. Anesthesia continued for 10.3 h. During the procedure, hemodynamic lability was observed, predominantly during emergence, with systolic pressures ranging from 79 to 170 mmHg. Perioperative ceftriaxone and dexamethasone were administered. The patient received 2500 mL of normal saline and 2000 mL of albumin on the day of the procedure. The day following the procedure, the serum bicarbonate had declined from 28 to 23 mmol/L (Fig. 1), and the serum creatinine was unchanged at 0.9 mg/dL. Urine dipstick revealed glycosuria (Table 1), though serum glucose ranged from 118 to 150 mg/dL. Four days later, this glycosuria declined to “trace.”

**Table 1:** Urine dip-stick results

	Day 1	Day 5	Day 8	Day 10	Day 14	Day 19	Day 21
pH	6.5	7.0	5.5–6.5	5.5	6.5	5.5	6.5
Glucose	1000	Trace	300–1000	150	Trace	Neg	Neg
Albumin	Neg	Neg	Neg-30	Trace	Trace	Neg	Neg
Ketones	Neg	Neg	1–4+	4+	Neg	Neg	Neg
Heme	Neg	Neg	Neg-trace	1+	Neg	Neg	Neg
LE	Neg	Neg	Neg	Neg	Neg	Neg	Neg
Nitrite	Neg	Neg	Neg	Neg	Neg	Neg	Neg

Glucose concentrations are estimates in mg/dL. Ranges on day eight occur due to duplicate urinalyses sent. The patient underwent surgery on days *zero*, *six*, and *nine*. LE: leukocyte esterase. Urine chemistries were analyzed on a Clinitec Atlas Urine Analyzer (Siemans Corp. Malvern, PA). A urine glucose measurement of 1000 mg/dL with this instrument was confirmed to be between 750 and 1500 mg/dL with comparator instruments 88–90% of the time (Chien et al. 2007).

Six days after the first procedure, the patient underwent a 3.25-h surgical exploration for cerebrospinal fluid leak. Anesthesia resembled the previous procedure except that the patient received sevoflurane at a fresh gas flow rate of 2 L/min, with concentrations ranging 1.75–2.25%. Hemodynamics resembled the first procedure, and the patient again received dexamethasone but did not receive additional ceftriaxone.

On the eighth day, urine dipstick again showed an estimated glucose of over 1000 mg/dL and ketonuria, in the context of serum glucose of 116 mg/dL. A confirmatory urinalysis was similar, and Nephrology was consulted. Nine days after the initial surgery, a third five-hour neurosurgical procedure was performed using desflurane. Prior to surgery, the patient’s serum bicarbonate was 30 (an arterial blood gas drawn two hours earlier and prior to extubation showed pH 7.47, pCO_2_ 41 mmHg, consistent with metabolic alkalosis with little respiratory compensation). After the surgery, the serum bicarbonate declined to 25 mmol/L (with arterial pH 7.46 and pCO2 37 mmHg three hours after extubation, showing some correction of the metabolic alkalosis). The patient experienced no gastrointestinal losses during this period, but was receiving intravenous normal saline. The serum phosphate declined from 2.7 to 1.7 mg/dL the next morning (Figure 1). A 24-h urine collection revealed phosphorus excretion of 1496 mg, with a 42% fractional excretion. Serum potassium declined from 4.1 to 3.2 mEq/L, with a 24-h potassium excretion of 60 mEq. The 24-h urinary protein was 406 mg, with no detectable albumin (Table 2). Serum creatinine remained stable at roughly 0.6–0.7 mg/dL. The patient remained hemodynamically stable but required ventilator support for 2 days. The patient’s mental status improved incrementally over the next 2 weeks. Twenty-one days following the first procedure, the patient’s creatinine remained unchanged, and urine studies revealed no glycosuria, a urine protein/creatinine of 0.06 g/g, and the fractional excretion of phosphorus that had declined to 10.7%.

**Table 2:** 24-h urine collection (Day 12)

Volume	2.9 L
Creatinine	1421 mg
Phosphate	1496 mg
Potassium	60 mEq
Protein	406 mg
Albumin	Undetectable

The patient received 16 mmol of enteral phosphate salts just before the 24-hour urine collection, bringing the serum phosphate level from 1.7 mg/dL the night before the collection to 1.9 just before the collection. Further repletion was deferred during urine collection. Fractional excretion of phosphorus was 42%.

## Discussion

Several findings in this patient are consistent with proximal tubule impairment. The patient exhibited marked glycosuria in the context of minimally elevated serum glucose levels. Hypophosphatemia accompanied urinary wasting of phosphate wasting. Mild proteinuria without albuminuria suggested reduced proximal protein reabsorption without glomerular damage. Ketonuria in the absence of hypoglycemia suggested reduced reabsorption of filtered organic acids. The patient exhibited inappropriately elevated urinary potassium excretion and a decline in serum bicarbonate (however, administration of chloride-rich intravenous fluids may have contributed to both of these findings, and the decline in bicarbonate could be interpreted as appropriate correction of metabolic alkalosis). Taken together, this patient’s glycosuria, phosphate wasting, nonalbuminuric proteinuria, ketonuria, and kaliuresis suggest impaired proximal tubule function. The repeated occurrence of these findings following surgery implicates a feature of the patient’s perioperative care in these findings.

Several features of proximal tubule impairment have been described in association with exposure to volatile anesthetics. Mean daily glucose excretion following administration of volatile anesthetics is elevated, and has been reported to vary in different subjects, from around 200 mg to as much as 6 g (Ebert et al. 1998; Goldberg et al. 1999; Obata et al. 2000; Higuchi et al. 2001; Kharasch et al. 2001), with some individuals exceeding 10 g (Kharasch et al. 1997, 2001). Glycosuria following volatile anesthetic exposure is transient, with levels often peaking in 1–5 days (Kharasch et al. 1997; Goldberg et al. 1999; Ebert and Arain 2000) and resolving within seven to 14 days (Eger et al. 1997b; Ebert et al. 1998; Goldberg et al. 1999). Glycosuria is seen in healthy research volunteers (Eger et al. 1997b; Ebert et al. 1998; Goldberg et al. 1999) in addition to surgical patients, suggesting that the observed glycosuria is related to anesthesia rather than clinical confounders such as may occur in surgical patients. Glycosuria has been frequently observed following the use of sevoflurane (Eger et al. 1997b; Kharasch et al. 1997, 2001; Goldberg et al. 1999; Groudine et al. 1999; Obata et al. 2000; Conzen et al. 2003) and isoflurane (Kharasch et al. 1997; Groudine et al. 1999; Obata et al. 2000; Conzen et al. 2003). One report showed only mild glycosuria following desflurane (Eger et al. 1997a) whereas another found none (Eger et al. 1997b), suggesting that this agent may have reduced glycosuric effects compared to other volatile anesthetic agents.

Proteinuria has been observed following the use of sevoflurane, isoflurane, and desflurane (Ebert and Arain 2000; Obata et al. 2000; Kharasch et al. 2001). Proteinuria varies widely between individuals, but often occurs in the range of 1 g per day, seldom reaching nephrotic levels. Studies that specify urinary albumin differ on the degree of albuminuria, but it often appears to be significantly less than the total protein excreted (Eger et al. 1997a; Ebert et al. 1998; Obata et al. 2000). As with glycosuria, proteinuria is observed in nonsurgical volunteers (Eger et al. 1997a,b; Ebert et al. 1998; Goldberg et al. 1999). The time-course of proteinuria parallels that of glycosuria, typically abating within 7 days (Eger et al. 1997b; Higuchi et al. 1998; Goldberg et al. 1999). As with glycosuria, desflurane appears to induce less proteinuria than sevoflurane (Eger et al. 1997a).

Proteinuria could be an indication of glomerular damage, however the observation that proteinuria following anesthetic exposure is often largely nonalbuminuric in nature is more suggestive of proximal tubule dysfunction. A better indicator of proximal tubule impairment is urinary excretion of *β*-2-microglobulin, a small, freely-filterable protein that is normally completely reabsorbed in the proximal tubule (Bagshaw et al. 2007). Exposure to either isoflurane or sevoflurane causes urinary elaboration of *β*-2-microglobulin (Higuchi et al. 1998), suggesting reduced proximal tubule function. Quantitation of another urinary biomarker, *α*-glutathione-S-transferase (*α*-GST) also examines proximal tubule injury. Proximal tubule cells express this protein, and its extrusion into the urine heralds damage to these cells (Harpur et al. 2011). A urinary *α*-GST spike occurs following exposure to sevoflurane (Eger et al. 1997a,b; Ebert et al. 1998; Goldberg et al. 1999), but not desflurane (Eger et al. 1997a,b).

Taken together, the above studies suggest that transient proximal tubule impairment following exposure to volatile anesthetics may be relatively common. Direct examination of this hypothesis by quantitation of markers of proximal tubule function, such as urinary excretion of organic acids, phosphate, potassium, and bicarbonate has not been reported. Although these observations suggest impairment of proximal tubule function, these effects are consistently transient, and these findings do not indicate renal tubular injury, kidney tubule injury, *per se* (Kharasch et al. 2001). Numerous reports indicate that these agents cause no detriment in glomerular filtration (Bito and Ikeda 1996; Bito et al. 1997; Kharasch et al. 1997, 2001; Ebert et al. 1998; Higuchi et al. 1998, 2001; Nishiyama et al. 1998; Bito et al. 1999; Goldberg et al. 1999; Groudine et al. 1999; Ebert and Arain 2000; Eger et al. 1997a,b; Obata et al. 2000; Conzen et al. 2002; Nishiyama 2013), suggesting that they do not induce necrosis of tubule cells in humans. On the contrary, previous reports have shown that volatile anesthetics protect proximal tubule cells against ischemia (Zager et al. 1999) and protect patients against ischemic nephropathy (Cai et al. 2014; Fukazawa and Lee 2014). Desflurane, which is less protective than other agents (Lee et al. 2004), is also associated with milder features of proximal tubule impairment (Eger et al. 1997a,b), suggesting that volatile anesthetic-associated proximal tubule impairment and protection against ischemic nephropathy may share a common mechanism. An intriguing hypothesis that could explain both phenomena is that volatile anesthetics decrease metabolic activity in proximal tubule epithelial cells, leading to transient proximal tubule impairment but reducing oxygen demand and the risk of acute tubular necrosis. In support of this hypothesis, exposure to isoflurane has recently been shown to reduce kidney deoxyhemoglobin levels, consistent with decreased oxygen consumption, although increased perfusion could not be excluded as an alternate explanation of this finding (Niles et al. 2015).

The absence of reports linking volatile anesthetics with frank Fanconi syndrome, as characterized by proximal renal tubular acidosis with significant hypokalemia and hypophosphatemia, suggests that proximal tubule dysfunction occurring after volatile anesthetic exposure is likely to be too mild or transient to cause significant harm to most patients. If the effects of volatile anesthetics on proximal tubule function can be confirmed on a more general group of patients, an interesting question will be which patients are most sensitive to these effects, whether there is any cumulative effect of repeated exposure to volatile anesthetics, and whether these agents cause more clinically significant features of proximal tubule dysfunction in patients predisposed to proximal tubule injury, such as those with multiple myeloma or receiving other medications known to be toxic to the kidney’s proximal tubule.

## Conflicts of Interest

None declared.
